# TRMT10C promotes mitochondrial fission through transferrin receptor m1A methylation modification and aggravates the progression of atrial fibrillation

**DOI:** 10.1002/ccs3.70059

**Published:** 2026-06-10

**Authors:** Nijina Li, Hao Zhang

**Affiliations:** ^1^ Department of Anesthesiology The Second Xiangya Hospital, Central South University Changsha Hunan China; ^2^ Department of Cardiovascular Surgery The Second Xiangya Hospital, Central South University Changsha Hunan China

**Keywords:** atrial fibrillation, atrial fibrosis, m1A methylation, mitochondrial fission, TRMT10C

## Abstract

Atrial fibrillation (AF) is a common cardiac arrhythmia often accompanied by structural remodeling of the atria, particularly fibrosis, and disruption of normal mitochondrial function. N(1)‐methyladenosine (m1A), a methylation of RNA, is gaining attention for its role in diverse biological processes. This study aimed to explore the role of the m1A methyltransferase tRNA methyltransferase 10C (TRMT10C) in AF pathogenesis. In the study, TRMT10C and m1A methylation levels were upregulated in AF rats, accompanied by excessive mitochondrial fission and myocardial fibrosis. Knockdown of TRMT10C inhibited the expression levels of mitochondrial fission‐related proteins Drp1 and Fis1, reduced collagen deposition (collagen I, Postn, collagen III, and fibronectin), and AF progression. In vitro results showed that TRMT10C knockdown inhibited TGF‐β1‐induced cardiac fibroblasts proliferation and migration, whereas overexpression of transferrin receptor (TFRC) reversed this effect. Mechanistically, TRMT10C enhanced the stability of TFRC mRNA by promoting m1A methylation, driving mitochondrial fission and fibrosis. Collectively, our findings elucidate a novel TRMT10C–TFRC m^1^A axis driving pathological mitochondrial dynamics and fibrosis in AF, offering new insight into cell‐signaling pathways underlying atrial disease and potential therapeutic targets.

## Graphical abstract

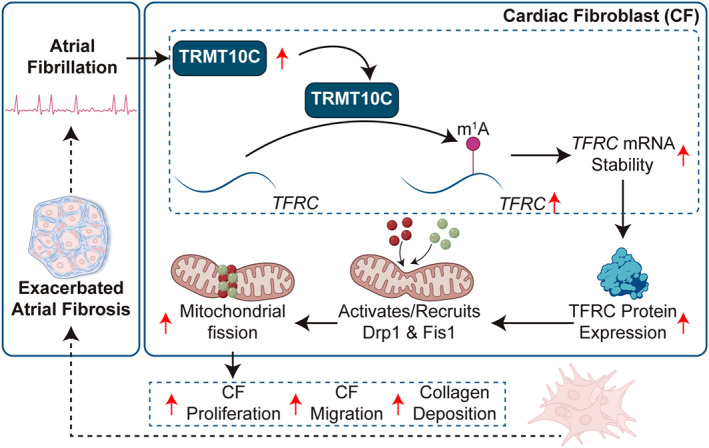

## 1 INTRODUCTION

Atrial fibrillation (AF) is the most commonly seen arrhythmia with a high risk of death and disability.^1^, ^2^ The most typical structural change in AF progression is atrial fibrosis, an important pathological factor that promotes AF and has great diagnostic and therapeutic significance.^3^, ^4^, ^5^ However, current research on the mechanism of atrial fibrosis has not yet provided effective clinical intervention targets. Therefore, comprehensive research on the core mechanism of atrial fibrosis is required in order to bring about breakthroughs in clinical treatment.^6^

Myocardial fibrosis acts as a crucial pathological change within the development of cardiovascular disease, which includes atrial fibrosis and myocardial fibrosis in other parts of the heart.^7^ Myocardial fibrosis is marked by the capacities of cardiac fibroblasts (CFs) to proliferate and to migrate and following collagen accumulation.^8^, ^9^, ^10^ This capacity is driven via mitochondrial metabolic reprogramming, including mitochondrial fission. In fact, abnormal mitochondrial fission might impede cellular activities, such as the capacity of cells to proliferate and to migrate.^11^, ^12^, ^13^ Mitochondrial fission is modulated via mitochondrial fission factor, mitochondrial fission proteins MID49, MID51, dynamin‐related protein 1 (Drp1), and mitochondrial fission 1 (Fis1) proteins.^14^, ^15^, ^16^ However, it remains unclear if mitochondrial fission serves as a pivotal factor within CF proliferation and migration, as well as its functional role within atrial fibrosis.

Dynamic and reversible chemical modifications of RNA exert a crucial effect on posttranscriptional gene regulation.^17^ More than 100 posttranscriptional RNA modifications have been discovered, such as N6‐methyladenosine (m6A), 5‐methylcytidine (m5C), N1‐methyladenosine (m1A), and pseudouridine, which exert key effects on cell differentiation, protein production, and biological modulation.^18^, ^19^, ^20^ A methyl group was attached to the N1 site of adenosine to produce m1A. Because of the methyl group and positive alteration within m1A, it could remarkably change the RNA structure and the strength of protein–RNA interactions.^21^, ^22^ m1A methylation regulators consist of “writers” (TRMT10C, TRMT61B, TRMT6, TRMT61A), “erasers” (ALKBH1, ALKBH3), and “readers” (YTHDF1, YTHDF2, YTHDF3, YTHDC1).^23^ The latest study shows that knocking out METTL3 prevents pathological mitochondrial fission and improves inflammation and myocardial fibrosis in ischemia‐reperfusion mice.^24^ Similarly, studies have shown that elevated METTL3 levels promote mitochondrial fission and elevated myocardial fibrosis within cardiac tissue of AF patients.^12^ Based on this speculation, post‐transcriptional RNA modification is associated with mitochondrial dynamics imbalance, myocardial fibrosis and AF progression. However, under AF conditions, the “landscape” and potential role of m1A‐based mRNA modification are still unclear.

Based on this, this study mined related differentially expressed genes through bioinformatics, selected the m1A modification “writers” gene TRMT10C through expression differences, and screened the transferrin receptor (TFRC) factors regulated by it. It was proposed that TRMT10C promotes mitochondrial dynamics imbalance (excessive fission) by regulating TFRC m1A methylation modification, induces myocardial fibrosis, and exacerbates the progression of AF, and verified it through in vivo and in vitro experiments.

## 2 MATERIALS AND METHODS

### 2.1 Animal grouping and treatment

Sprague–Dawley rats (male, 200–250 g, 8‐week‐old, *N* = 30) were collected from Hunan Silaike Jingda Laboratory Animal Co., Ltd. The rats were housed in a specific pathogen free animal room (12 h light/12 h dark cycles, 20–25°C, 50%–60% humidity) and had access to food and water ad libitum. The study was carried out under the approval of the Animal Ethics Committee of The Second Xiangya Hospital, Central South University (Approval No. 20240175).

The rats were randomized into five groups (five rats for each): Control group; AF group; AF + AAV‐shNC group; AF + AAV‐shTRMT10C 1# group; AF + AAV‐shTRMT10C 2# group. Normal controls received injection with 0.9% saline at a dose of 1 mL/kg via the tail vein every day for 7 days. The rats in the AF group were injected with acetylcholine (ACh)‐CaCl_2_ (60 μg/mL ACh and 10 mg/mL CaCl_2_) at 1 mL/kg via the tail vein every day for 7 days. The rats in the AAV‐NC group, AF + AAV‐shTRMT10C 1# group and AF + AAV‐shTRMT10C 2# group received injection with AAV‐NC or AAV‐shTRMT10C 1# or AAV‐shTRMT10C 2# (2 × 10^11^ vector genome [vg] particles/per rat) via tail vein, and the modeling operation was the same as that of AF group 14 days after the AAV injection.^25^

### 2.2 Isolation of CFs cells

CFs were isolated from neonatal rat hearts. Briefly, the hearts of neonatal rats (2–3 days old) were rapidly excised. After washing in pre‐cooled PBS solution, the heart tissues were sheared and digested in 37°C for 7‐to‐8 min. The digestion enzymes included trypsin (0.25%, 15050065, Thermo Fisher Scientific) and collagenase type II (0.67%, 17101015, Thermo Fisher Scientific). After the tissues began to loosen, pre‐cooled Dulbecco's modified eagle medium (DMEM) media (10% FBS, Gibco) was supplemented to halt the digestion. The cells extracted from the tissues were harvested, followed by 120‐min culture within DMEM media (glucose 5.5 mM) added with 10% FBS (37°C, 5% CO_2_). Postn (sc‐398631 FITC, Santa Cruz Biotechnology) positive cells (cell sorting) were regarded as CFs. Upon reaching 80% confluence, cells were passaged at a ratio of 1:1, and the cells from the second to fourth passage were employed for subsequent analyses. CF cells were subjected to 24‐h treatment with 50 ng/mL TGF‐β1 to induce a cell model.

### 2.3 Cell transduction

TRMT10C knockdown or TFRC overexpression in primary rat CFs was achieved by transfecting vector contained short hairpin RNA targeting TRMT10C (target site 1: GCCAGATATCTGGTGCCATAT; target site 2: GCTGCAACCAGAGAGCTAATT; sh‐TRMT10C #1, sh‐TRMT10C #2; sh‐NC) or plasmid overexpressing TFRC (OE‐TFRC). The vector containing scramble sequence short hairpin RNA or empty expression vector was set as negative control (sh‐NC, OE‐NC) for the knockdown vector and overexpression vector. OriTrans®PEI‐DNA (ORI2305, Ori‐Bio) was utilized to perform transfection as per the instructions of the manufacturer, followed by 48‐h incubation. Subsequently, cells were harvested for further analysis.

### 2.4 Electrocardiograms

After experimental treatment, electrocardiogram (ECGs) on rats were recorded under anesthesia. 30‐gauge subcutaneous needle electrodes (Grass Technologies) and the BL‐420F bio‐function experiments system (Chengdu Techman Software Co., Ltd.) were employed to assess ECGs, thereby capturing standard lead II ECG traces. According to protocols described in a prior study,^26^ after the last ACH‐CaCl2 solution injection, ECG signals were recorded. The induction of AF was confirmed by P wave disappearance, erratic heartbeats, and variable R‐R intervals on ECG, indicating that a rat AF model was successfully established.^27^ The AF duration time was recorded.

### 2.5 AERP evaluation

Following AF induction, a 2.0 F electrophysiological catheter (Abbott Molecular) was inserted to the right atrium via the jugular vein. Atrial effective refractory period (AERP) was determined using the S1–S2 programmed electrical stimulation technique (2X diastolic threshold; duration, 2 ms), employing the S2 extra‐stimulus method with 8 consistent stimuli. AERP was identified as the longest S1–S2 interval failing to provoke an atrial depolarization, and the measurement of AERP with a basic drive cycle length of 150 ms was conducted. The AERP times were recorded. Subsequently, after euthanizing rats, atrial tissue samples were either kept at −70°C or fixed with 4% paraformaldehyde at 4°C for future experiments.

### 2.6 Transmission electron microscope

According to previous research methods,^28^ atrial tissue was cut into 1 mm^3^ pieces, pretreated with 2% glutaraldehyde, and subjected to fixation with 1% osmium tetroxide. Next, the specimens were subjected to dehydration using 3% uranyl acetate in ethanol and then embedded within epoxy resin and propylene oxide for overnight polymerization. They were sectioned into 70‐nm thick slices and subjected to staining with lead citrate and then examined with a transmission electron microscope (HT7800, Hitachi).

### 2.7 Histopathological examinations

As for hematoxylin and eosin extracellular matrix (ECM) staining, atrial tissue samples were subjected to fixation with 4% paraformaldehyde for one night, paraffin‐embedded, and subsequently sectioned into slices at a thickness of 5 μm as previously described.^29^ After being deparaffinized, slices were subjected to 5‐min staining at room temperature (RT) with hematoxylin (H8070, Solarbio). Slices were washed in distilled water, followed by 3‐min counterstaining at RT with eosin. An Olympus Corporation BX53 light microscope was applied to observe the pathological alterations.

As for Masson's staining, the slices were subjected to Masson's Trichrome Stain kit (G1340, Solarbio) followed the manufacturer's instructions. After staining, collagen and mucin will be colored blue. The atrial fibrosis degree was observed under light microscope.

### 2.8 CCK‐8 assay

The cell suspension (5 × 10^3^ cells/100 μL/well) was seeded onto a 96‐well plate and the plate was subjected to 24‐h pre‐culture within an incubator. After treatment according to the groups, each well was supplemented with 10 μL of CCK‐8 solution (C0038, Beyotime), followed by 2‐h incubation in an incubator. A microplate reader (BioTek) was used to assess the absorbance at 450 nm.

### 2.9 EdU assay

Cell proliferation was detected according to the method of the kit (C10310‐1, RiboBio). After transfection as described above, the cells were placed into six‐well plates, followed by 24‐h treatment with 50 ng/mL TGF‐β1. EdU working solution (final concentration of EdU was 10 μM) preheated at 37°C was supplemented, followed by 2‐h incubation. Following EdU labeling of cells, the culture medium was removed, cells were subjected to 15‐min fixation with 4% paraformaldehyde at RT, washed in sequence, and permeabilized with 0.3% TritonX‐100. Next, the click reaction solution was supplemented, followed by 30‐min incubation at RT away from light. 4'6‐diamidino‐2‐phenylindole was used for cell nucleus staining, and a fluorescence microscope was applied to observe and photograph cells.

### 2.10 Wound healing assay

Rat CFs were cultured until the cells formed a confluent monolayer. A scratch (about 0.5–1 mm) was made on the cell monolayer perpendicular to the bottom of the culture plate. Following the scratch, cells were rinsed in serum‐free media to remove cell debris, and cells were maintained in culture. The migration of cells to the scratch area was observed under a microscope and photographed at 0 and 48 h.

### 2.11 Mitochondrial labeling

MitoTracker Red CMXRos probe (C1049B‐50 μg, Beyotime) was employed to label cell mitochondria. Cells were subjected to 30‐min incubation at 37°C with MitoTracker Red, and rinsed two times in PBS. A fluorescence microscope was employed to capture images. Fluorescence intensity was assessed with excitation/emission settings at 579/599 nm, and the mitochondrial length was calculated according to the fluorescence signals.

### 2.12 Real‐time quantitative PCR

TRIzol reagent (15596018CN, Invitrogen) was utilized to extract total RNA from rat atrial tissues or CFs, and High‐Capacity cDNA Reverse Transcription Kit (4368814, Thermo Fisher Scientific) was used to reverse transcribe RNA into cDNA. Real‐time quantitative PCR (qRT‐PCR) was conducted upon an ABI 7500 Real‐Time PCR system using SYBR Green PCR Master Mix (4309155, Thermo Fisher Scientific). The 2^−ΔΔ^Ct method was applied to calculate relative mRNA levels (sequence details provided in Table S1).

### 2.13 Methylated RNA immunoprecipitation followed by qRT‐PCR

GenSeq m1A MeRIP kit (GS‐ET‐002, CloudSeq Biotech) was used as per the protocols of the manufacturer. RNA fragmentation reagent was utilized to fragment the extracted total RNA from human CFs (Procell, Wuhan, China) to ∼100 nucleotides. m1A‐modified RNA fragments were immunoprecipitated using anti‐m1A antibody bound to Protein A/G magnetic beads. After washing, methylated RNA was eluted, purified, and reverse transcribed. qPCR was performed using TFRC‐specific primers. The relative m1A enrichment was calculated as the percentage of input RNA.

### 2.14 RNA stability assay

To evaluate the effect of TRMT10C upon TFRC mRNA stability, RNA decay assays were performed. CFs were stimulated with actinomycin D (Act‐D, 5 μg/mL; Cat. #sc‐200906, Santa Cruz Biotechnology) at 48 h post‐transfection. Cells were collected at 0, 2, 4, and 6 h following Act‐D treatment, and TRIzol was applied to extract total RNA. cDNA was synthesized and used for qRT‐PCR quantification of TFRC mRNA. The remaining mRNA level at each time point was normalized to 0 h, and mRNA half‐lives were calculated using a first‐order decay model.

### 2.15 Dot blot assay

TRIzol reagent (Invitrogen) was employed to extract total RNA, which was then diluted with 10 mM Tris‐EDTA buffer after quantification. Equal amounts of RNA specimens were loaded on Amersham Hybond‐N+ membranes (GE Healthcare). After UV crosslinking, the membranes were subjected to 1‐h blocking with 5% milk, an overnight incubation at 4°C with anti‐m1A antibody (ab208196, Abcam), and then 1‐h incubation at RT with HRP‐labeled anti‐rabbit IgG (Cell Signaling Technology), and lastly assessed with ECL SuperSignal Blotting Detection Reagent (Thermo Fisher Scientific).

### 2.16 Western blot

Proteins were isolated from rat atrial tissue samples or CFs. A BCA kit (P0009, Beyotime) was employed to measure protein contents. Proteins (30 μg/sample) were subjected to 10% SDS‐PAGE electrophoresis at 80 V for 2.5 h, followed by polyvinylidene fluoride membrane transformation (LC2005, Thermo Fisher Scientific). Membranes were subjected to 1‐h blocking at RT with 5% BSA (BL2182A, Biosharp), followed by an overnight incubation at 4°C with primary antibodies against TRMT10C (29087‐1‐AP, Proteintech), ALKBH3 (12292‐1‐AP, Proteintech), Collagen I (AF7001, Affinity), Postn (66491‐1‐Ig, Proteintech), Collagen III (22734‐1‐AP, Proteintech), Fibronectin (AF5335, Affinity), Drp1 (12957‐1‐AP, Proteintech), Fis1 (10956‐1‐AP, Proteintech), TFRC (ab214039, Abcam) or m1A (68636‐1‐Ig, Proteintech). After 2‐h incubation at RT with secondary antibodies and rinsing with Tris‐buffered saline with Tween 20, an automatic imaging system (Tanon‐5200, Tanon) with ECL solution (P0018M, Beyotime) was employed to visualize protein bands.

### 2.17 GO enrichment analyses

The intersecting genes were subjected to gene ontology (GO) enrichment analyses. The Database for KEGG orthology based annotation system (KOBAS, http://bioinfo.org/kobas/) was utilized to conduct these analyses.^30^ Cutoffs were set at FDR < 0.05 and *p* < 0.05. Subsequently, the results were visualized with the use of R version 3.4.1.

### 2.18 Statistical analysis

All data were analyzed using GraphPad Prism 9.0 software (GraphPad Software). Before statistical testing, data distribution normality was assessed using the Shapiro–Wilk test. For normally distributed data, comparisons among groups were assessed using one‐way analysis of variance with Tukey's post hoc test, and comparison between groups were conducted using Student's *t*‐test. In cases where the data did not meet the assumptions of normality, the Kruskal–Wallis test with Dunn's post hoc test was applied. All data were represented in terms of mean ± standard deviation. Significance was determined at probability levels of *p* < 0.05.

## 3 RESULTS

### 3.1 TRMT10C‐mediated mitochondrial dynamics imbalance may be a crucial factor in the progression of AF

**FIGURE 1:**
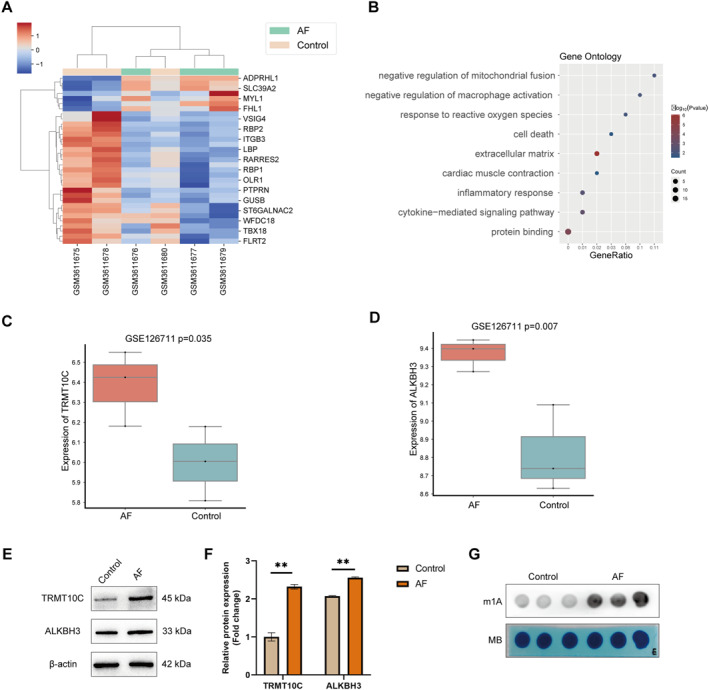
Bioinformatics analysis of differentially expressed genes in atrial fibrillation. (A) Mining differentially expressed genes based on the atrial fibrillation chip GSE126711; (B) KOBAS analysis of differentially expressed genes; (C, D) Analysis of the expression of m1A modification regulatory factors (TRMT10C, ALKBH3) based on the atrial fibrillation chip GSE126711; (E, F) Western blot detection of the expression of TRMT10C and ALKBH3 in rat atrial tissue; (G) Dot blot detection of the m1A methylation modification level in rat atrial tissue. (*N* = 3), **p < 0.01.

Based on the GEO database, the AF chip GSE126711 was mined, which included 3 rat AF samples repeatedly induced after 2 days of continuous RAP and 3 controls.^31^ Using limma differential analysis, 33 differential genes were screened (|log2FC| > 1.2, *p*.value < 0.05), consisting of 7 increased genes and 26 decreased genes (Figure 1A). KOBAS analysis of 33 target genes with significant differences found that they were significantly associated with biological processes such as ECM, protein binding, cytokine‐mediated signaling pathways, inflammatory response, negative modulation of mitochondrial fusion, negative modulation of macrophage activation, reactive oxygen species response, cell death, and myocardial contraction (Figure 1B). Based on the literature, we obtained 10 m1A modification regulators, including “writers” (TRMT10C, TRMT61B, TRMT6, TRMT61A), “erasers” (ALKBH1, ALKBH3) and “readers” (YTHDF1, YTHDF2, YTHDF3, YTHDC1)^23^; and the enhanced expression of TRMT10C protein led to impaired mitochondrial function,^32^ so it is speculated that the imbalance of mitochondrial dynamics mediated by TRMT10C may be the key cause of AF. In GSE126711, only TRMT10C and ALKBH3 were significantly upregulated in AF group (Figure 1C,D). As revealed by Western blot, TRMT10C and ALKBH3 were increased in AF group (Figure 1E,F). As shown by Dot blotting, the m1A methylation modification level within the atrial tissues of AF group was upregulated (Figure 1G).

### 3.2 Knockdown of TRMT10C inhibits mitochondrial fission, atrial fibrosis, and progression of AF

**FIGURE 2:**
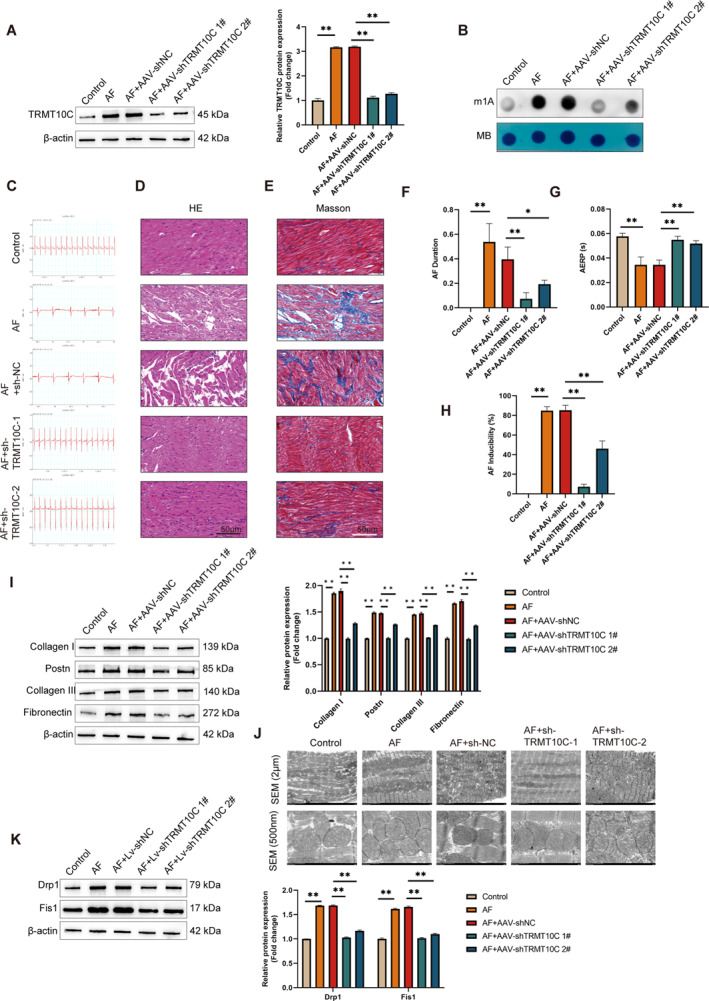
Knockdown of TRMT10C inhibits mitochondrial fission, atrial fibrosis, and progression of atrial fibrillation. The rats were divided into Control, AF, AF + AAV‐shNC, AF + AAV‐shTRMT10C 1#, AF + AAV‐shTRMT10C 2#. (A) Western blot detection of the expression of TRMT10C in rat atrial tissue; (B) dot blot detection of the m1A methylation modification level in rat atrial tissue; (C) aurface electrocardiogram of rats; (D) HE staining to evaluate the left atrium, Scale bar: 50 μm; (E) Masson trichrome staining to evaluate the left atrium, Scale bar: 50 μm; (F) AF duration; (G) evaluation of rat AERP; (H) evaluation of AF susceptibility statistics; (I) Western blot detection of collagen I, Postn, collagen III and fibronectin protein expression levels; (J) Electron microscopy to detect mitochondrial fission; (K) Western blot detection of mitochondrial fission‐related proteins Drp1 and Fis1 expression levels. (*N* = 3 or 6), *p < 0.05, **p < 0.01. AERP, atrial effective refractory period; AF, atrial fibrillation.

Rats were injected with AAV via tail vein to knock down TRMT10C to evaluate the effect of TRMT10C on mitochondrial fission, atrial fibrosis, and progression of AF. As demonstrated by Western blot, TRMT10C protein was upregulated within the AF group, and the TRMT10C protein level was successfully downregulated after injection of AAV (AF + AAV‐shTRMT10C 1#, AF + AAV‐shTRMT10C 2#), and the knockdown efficiency was higher in the AF + AAV‐shTRMT10C 1# group (Figure 2A). The dot blot was employed to detect the m1A methylation modification level, indicating that the increased m1A methylation modification level in the AF group was synchronously reduced after AAV knockdown of TRMT10C (Figure 2B). The surface ECG of rats showed that the P wave duration and interval were longer within the AF group, and the P wave duration and interval were slowed down after knockdown treatment (Figure 2C). HE staining and Masson staining revealed that the myocardial fibers of rats within normal controls were neatly arranged and bundled, the myocardial cells and nuclei were of normal size, and there was no tissue vacuolation, tissue edema, or inflammatory infiltration; the myocardial infarction area of the AF group was loose and edematous, with inflammatory cell infiltration, and the further necrotic muscle fibers were replaced by loose fibrous connective tissue, the scattered surviving myocardial cells at the edge of the infarction were hypertrophic, fibroblasts proliferated, collagen fibers were deposited in a large area, myocardial fiber cells were arranged in disorder, and some myocardial cells showed vacuolar degeneration. The pathological state was improved after knockdown treatment (Figure 2D,E). The AF group had prolonged AF duration, increased AF susceptibility, and shortened AERP, and these factors were improved after knockdown treatment (Figure 2F–H). Western blot detection of fibrosis‐associated proteins (collagen I, Postn, collagen III, and fibronectin) showed that fibrosis‐associated proteins were dramatically increased within the AF group, and significantly downregulated after knockdown treatment (Figure 2I).

Electron microscopy revealed distinct ultrastructural changes in the myocardium across the experimental groups. In the control group, myocardial fibers exhibited a well‐organized structure with a moderate number of mitochondria and minimal autophagy. The AF group presented with similarly clear myocardial fiber structure, but with an increased number of smaller mitochondria and the appearance of some vacuoles within the fibers, alongside a small amount of autophagy. Similarly, the AF + AAV‐shNC group displayed disordered and broken myocardial fibers, an increased quantity of diminished mitochondria, and some vacuolization, with autophagy remaining minimal. The AF + AAV‐shTRMT10C 1# group showed a return to a more normal phenotype, with clear myocardial fiber structure, a moderate mitochondrial population, and a small degree of autophagy. However, the AF + AAV‐shTRMT10C 2# group demonstrated the more severer pathology than shTRMT10C 1#; the myocardial fibers were disordered and fractured, mitochondrial size was reduced while their number increased, and some vacuolization was observed. This group was also characterized by a significant increase in autophagy, with some mitochondria appearing swollen and exhibiting fragmented or ruptured cristae (Figure 2J). The expression levels of mitochondrial fission‐associated proteins Drp1 and Fis1 were detected using Western blot, revealing that Drp1 and Fis1 were upregulated within the AF group and downregulated after AAV knockdown treatment (Figure 2K).

### 3.3 Knockdown of TRMT10C inhibits mitochondrial fission and fibrosis in atrial fibroblasts

**FIGURE 3:**
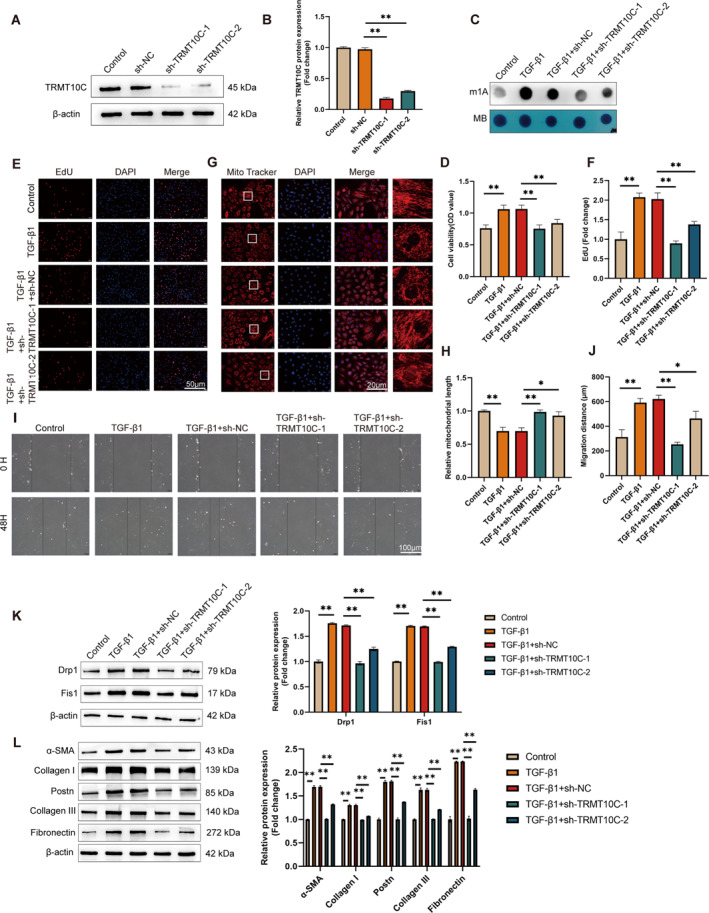
Knockdown of TRMT10C inhibits mitochondrial fission and fibrosis in atrial fibroblasts. CF cells were divided into Control, sh‐NC, sh‐TRMT10C‐1, sh‐TRMT10C‐2. (A, B) Western blot detection of TRMT10C protein knockdown efficiency. CF cells were divided into Control, TGF‐β1, TGF‐β1 + sh‐NC, TGF‐β1 + sh‐TRMT10C‐1, and TGF‐β1 + sh‐TRMT10C‐2. (C) Dot blot detection of the m1A methylation modification level in CF cell; (D) CCK8 detects cell viability; (E, F) EdU detects cell proliferation, Scale bar: 50 μm; (G, H) MitoTracker staining and statistics, Scale bar: 20 μm; (I, J) wound healing assay detects cell migration, Scale bar: 100 μm; (K) Western blot detection of mitochondrial fission‐related proteins Drp1 and Fis1 expression levels. (L) Western blot detection of α‐SMA, collagen I, Postn, collagen III and fibronectin protein expression levels. (*N* = 3), *p < 0.05, **p < 0.01. CF, cardiac fibroblast.

CF cells were extracted from rat tissue samples, and Postn‐positive cells were considered as CFs. TRMT10C was knocked down, and the effect of TRMT10C upon mitochondrial fission and fibrosis of CF cells was evaluated using the TGF‐β1‐induced mitochondrial hyperdifferentiation model of CF cells. As shown by Western blot, the shTRMT10C 1 and shTRMT10C 2 successfully downregulated the TRMT10C protein level, and the knockdown efficiency of the shTRMT10C 1# group was higher (Figure 3A,B). The dot blot was applied to detect the m1A methylation modification level, indicating that the increased m1A methylation modification level in the TGF‐β1 group was synchronously reduced after knocking down TRMT10C (Figure 3C). To detect cell viability and proliferation, CCK‐8 and EdU experiments were performed, suggesting that the TGF‐β1 group enhanced, whereas knocking down TRMT10C (TGF‐β1 + sh‐TRMT10C‐1, TGF‐β1 + sh‐TRMT10C‐2) repressed CF cell viability and proliferation (Figure 3D–F). Cell migration was detected using the wound healing. The results showed that TGF‐β1 group enhanced the migration ability of CF cells, whereas knocking down TRMT10C (TGF‐β1 + sh‐TRMT10C‐1, TGF‐β1 + sh‐TRMT10C‐2) inhibited the migration ability of CF cells (Figure 3I,J). To evaluate the mitochondrial status, MitoTracker staining and Western blot were performed to evaluate mitochondrial fission‐associated proteins Drp1 and Fis1, demonstrating that CF cell mitochondrial length was reduced and Drp1 and Fis1 expression was increased following stimulation with TGF‐β1 group. After knocking down TRMT10C (TGF‐β1 + sh‐TRMT10C‐1, TGF‐β1 + sh‐TRMT10C‐2), the mitochondrial length increased and Drp1 and Fis1 expression within CF cells were inhibited (Figure 3G,H,K). Finally, Western blot was conducted to detect fibrosis‐related proteins (type I collagen, Postn, type III collagen, and fibronectin), indicating that fibrosis‐associated proteins were dramatically increased within the TGF‐β1 group, and considerably decreased after knockdown of TRMT10C (TGF‐β1 + sh‐TRMT10C‐1, TGF‐β1 + sh‐TRMT10C‐2) (Figure 3L).

### 3.4 Bioinformatics analysis of factors regulated by TRMT10C

**FIGURE 4:**
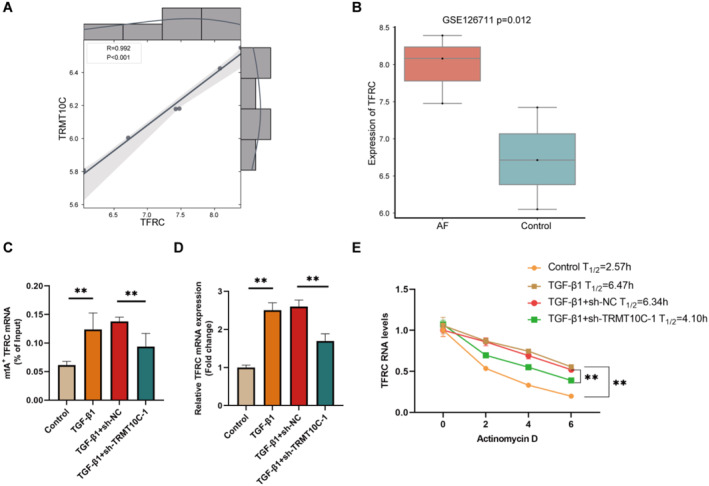
Bioinformatics mining of factors regulated by TRMT10C. (A) Pearson correlation analysis (TFRC, TRMT10C); (B) analysis of the expression of TFRC based on the atrial fibrillation chip GSE126711; (C) m1A MeRIP assay to detect the effect of TRMT10C knockdown on TFRC m1A methylation; (D) qPCR detection of the effect of TRMT10C knockdown on TFRC mRNA levels; (E) detection of TFRC mRNA stability by actinomycin D. (*N* = 3), **p < 0.01. TFRC, transferrin receptor.

Further Pearson correlation analysis was carried out upon the 33 differentially expressed genes screened within Figure 1A, and TFRC was found to be positively correlated with TRMT10C, and the correlation was the largest (Figure 4A). In GSE126711, TFRC in the AF group was significantly upregulated (Figure 4B). m1A methylated RNA immunoprecipitation (MeRIP) detected the effect of knocking down TRMT10C on TFRC m1A methylation. The results showed that after TRMT10C knockdown, the m1A modification level of TFRC mRNA decreased, indicating that TRMT10C may regulate TFRC through m1A modification (Figure 4C). qPCR detected the effect of knocking down TRMT10C on TFRC mRNA level, suggesting that TFRC expression increased after TGF‐β1 stimulation and was downregulated after knocking down TRMT10C (Figure 4D). TFRC mRNA stability was detected using Act‐D, suggesting that after TRMT10C knockdown, TFRC RNA stability decreased (Figure 4E).

### 3.5 TRMT10C promotes mitochondrial fission and fibrosis in CFs by promoting TFRC m1A methylation modification

**FIGURE 5:**
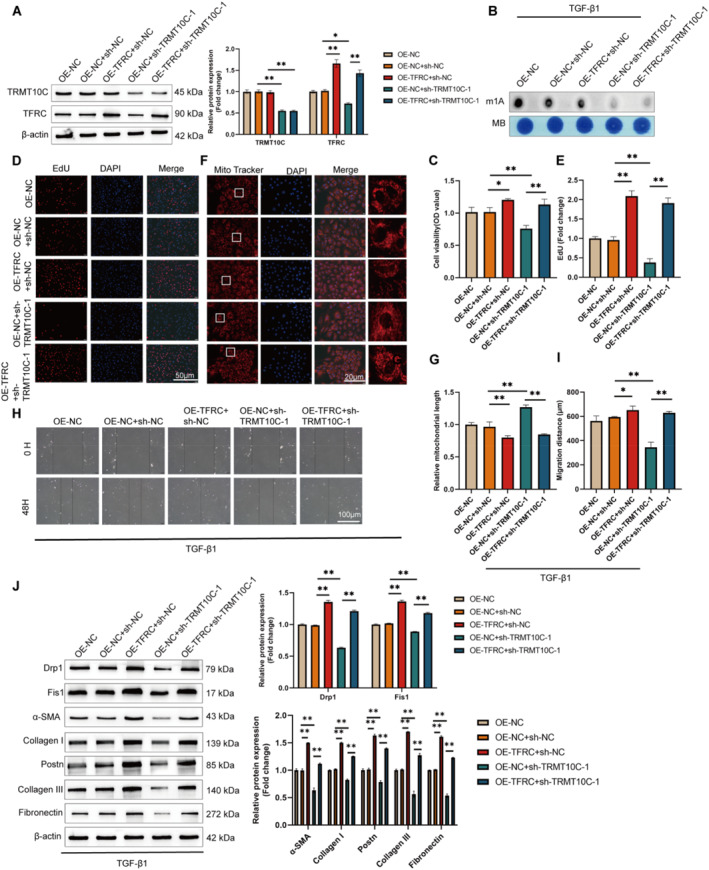
TRMT10C promotes mitochondrial fission and fibrosis in CFs by promoting TFRC m1A methylation modification. CF cells were pre‐treated with TGF‐β1 and divided into OE‐NC, OE‐NC + sh‐NC, OE‐TFRC + sh‐NC, OE‐NC + sh‐TRMT10C‐1, and OE‐TFRC‐sh‐TRMT10C‐1. (A) Western blot detection of TRMT10C and TFRC protein levels. (B) Dot blot detection of the m1A methylation modification level in CF cell; (C) CCK8 detects cell viability; (D, E) EdU detects cell proliferation, Scale bar: 50 μm; (F, G) MitoTracker staining and statistics, Scale bar: 20 μm; (H, I) wound healing assay detects cell migration, Scale bar: 100 μm; (J) Western blot detection of mitochondrial fission‐related proteins Drp1, Fis1 and fibrosis‐related proteins α‐SMA, collagen I, Postn, collagen III and fibronectin expression levels. *p < 0.05, **p < 0.01. CF, cardiac fibroblast; TFRC, transferrin receptor.

In order to explore whether TRMT10C plays a role in regulating TFRC, a plasmid was constructed to knock down TRMT10C and overexpress TFRC. As demonstrated by Western blot, in comparison with OE‐NC + sh‐NC and OE‐TFRC + sh‐NC, TRMT10C protein expression was downregulated in OE‐NC + sh‐TRMT10C‐1 and OE‐TFRC + sh‐TRMT10C‐1 groups; in comparison with OE‐NC + sh‐NC, TFRC protein expression was upregulated within OE‐TFRC + sh‐NC, TFRC protein expression was downregulated in OE‐NC + sh‐TRMT10C‐1, and TFRC protein expression was elevated within OE‐TFRC + sh‐TRMT10C‐1 compared with OE‐NC + sh‐TRMT10C‐1 (Figure 5A). This indicates that the expression of TFRC did not impact the expression of TRMT10C protein, whereas TRMT10C affects the expression of TFRC protein, and TFRC is a downstream factor of TRMT10C. Under TGF‐β1 stimulation, CFs cells were subjected to TFRC overexpression and TRMT10C knockdown. Then, the dot blot was used to detect the m1A methylation modification level, suggesting that the methylation modification level within the OE‐NC + sh‐TRMT10C‐1 and OE‐TFRC + sh‐TRMT10C‐1 groups was reduced in comparison with OE‐NC + sh‐NC and OE‐TFRC + sh‐NC, respectively (Figure 5B). Cell viability and proliferation were detected using CCK‐8 and EdU experiments, indicating that TFRC overexpression promoted, whereas TRMT10C knockdown repressed CF cell viability and proliferation (Figure 5C–E). The wound healing was used to assess cell migration ability, revealing that TFRC overexpression enhanced CF cell migration, whereas knockdown of TRMT10C inhibited the migration ability of CF cells (Figure 5H,I). To evaluate the mitochondrial status, MitoTracker staining was performed, demonstrating that after TFRC overexpression, CF cell mitochondrial length was shortened. After knockdown of TRMT10C, the mitochondrial length of CF cells increased (Figure 5F,G). The mitochondrial fission‐related proteins (Drp1, Fis1) and fibrosis‐related proteins (type I collagen, Postn, type III collagen, and fibronectin) were detected using Western blot. The results showed that mitochondrial fission‐associated proteins and fibrosis‐associated proteins were significantly upregulated after overexpression of TFRC, and mitochondrial fission‐associated proteins and fibrosis‐associated proteins were significantly downregulated after knocking down TRMT10C (Figure 5J). In the above experiments, overexpression of TFRC can antagonize all the effects caused by knocking down TRMT10C.

## 4 DISCUSSION

AF is one of the most commonly seen arrhythmias within clinical practice. Its pathogenesis is closely correlated with atrial fibrosis, and the core of atrial fibrosis lies in the abnormal proliferation, migration, and collagen deposition of CFs.^33^, ^34^ This study found that TRMT10C drives mitochondrial fission by regulating the m1A methylation modification of TFRC, thereby promoting atrial fibrosis and AF progression, providing a new perspective on the pathological mechanism of AF.

Atrial fibrosis, marked by the excessive activation of CFs and subsequent ECM deposition, provides the fundamental structural substrate for AF occurrence and persistence. Herein, TRMT10C expression showed to be increased within AF model rats' atrial tissue, accompanied by the activation of mitochondrial fission‐associated proteins Drp1 and Fis1, suggesting that abnormal mitochondrial fission might link TRMT10C and fibrosis. As previously reported, excessive mitochondrial fission could promote fibroblast activation by regulating cell metabolism and oxidative stress,^12^, ^35^ while inhibiting mitochondrial fission can alleviate myocardial fibrosis.^13^ A previous study demonstrated that METTL3 promotes mitochondrial fission through m6A modification.^24^ Notably, this study observed that knocking down TRMT10C could significantly inhibit TGF‐β1‐induced CF mitochondrial fission and reduce collagen deposition, suggesting that RNA methylation may serve as a common mechanism for regulating mitochondrial dynamic balance.

RNA methylation modification is a key regulatory mode of gene expression, and its role in cardiovascular diseases is gradually being revealed. In heart disease, METTL3‐mediated m6A modification can promote mitochondrial fission and fibrosis by regulating lncRNA GAS5.^24^ In recent years, other types of methylation have gradually been recognized. This study revealed that the m1A methylation level within AF rats' atrial tissue was increased, and TRMT10C, as a “writer” protein of m1A, could reduce the m1A modification level of TFRC when knocked down. As previously reported, m1A modification contributes to cell differentiation and the stress response by affecting mRNA stability and translation efficiency.^21^ In contrast, the regulation of TRMT10C‐mediated m1A modification on TFRC represents a new discovery in AF, but current research on the direct association between m1A modification and mitochondrial dynamic balance is limited, necessitating further exploration of the roles of other m1A regulatory factors in AF.

TFRC is a transmembrane glycoprotein extensively present on the cell membrane surface, and exerts a crucial effect on the process of cellular iron uptake.^36^ TFRC can specifically bind to transferrin and mediate the cell's iron uptake to meet the cell's iron needs for growth, proliferation, and maintaining normal physiological functions.^37^ TFRC is significantly upregulated in AF, and literature reports indicate that TFR1 knockout improves mitochondrial membrane potential, increases ATP content, downregulates mitochondrial fission proteins, and upregulates mitochondrial fusion proteins^38^; TFR1 is highly expressed in both coronary artery disease and AF.^39^ Given that the m1A methylation level within AF rats' atrial tissue is synchronously increased with TRMT10C, and TRMT10C is the “writer” protein of m1A, it was speculated that TRMT10C may mediate the m1A modification of TFRC, thereby impacting AF. This study confirmed that TRMT10C enhances TFRC mRNA stability by promoting m1A methylation, thereby driving mitochondrial fission. As a transferrin receptor, TFRC's function is mainly related to iron metabolism, but this study is the first to discover its role in the dynamic regulation of mitochondria in CF. Mechanistically, m1A modification may alter the secondary structure of TFRC mRNA, allowing it to evade nuclease degradation, which is similar to the pattern of YTHDF family proteins recognizing m1A sites and regulating mRNA stability.^40^ Additionally, overexpression of TFRC can reverse the repression of mitochondrial fission and the reduction of fibrosis caused by TRMT10C knockdown, suggesting that TFRC is a direct downstream target of TRMT10C.

This study first proposes the role of TRMT10C in AF by regulating TFRC m1A methylation modification, offering an underlying target for AF prevention and treatment. Targeting TRMT10C or TFRC m1A modification may become a new strategy to inhibit atrial fibrosis. In the future, the specific sites of TFRC m1A modification can be further clarified, and key modification sites can be located through methylation sequencing to more accurately regulate target genes and treat AF.

## AUTHOR CONTRIBUTIONS

Hao Zhang designed the experiments, project administration, drafted the article and revised the article critically for important intellectual content. Nijina Li contributed to cell and animal experiments. All authors read and approved the final manuscript.

## CONFLICT OF INTEREST STATEMENT

The authors declare no conflicts of interest.

## ETHICS STATEMENT

The study was carried out under the approval of the Animal Ethics Committee of The Second Xiangya Hospital, Central South University (Approval No. 20240175).

## CONSENT FOR PUBLICATION

Not applicable.

## Supporting information

Table S1

## Data Availability

All data generated or analyzed during this study are included in this published article (Table S1).
