# Supplementary material for: TRMT10C promotes mitochondrial fission through transferrin receptor m1A methylation modification and aggravates the progression of atrial fibrillation
Source: J Cell Commun Signal. 2026 Jun 10;20(2):e70059. doi: 10.1002/ccs3.70059 (PMC13251614; doi:10.1002/ccs3.70059)
Supplement: Supplementary file 1 — Table S1 [file CCS3-20-e70059-s001.docx]

**Table S1. The primer sequences for the qRT-PCR**

| **Gene** | **Primer** | **Sequence** **(5'-3')** |
| --- | --- | --- |
| TFRC | Forward | TGTGGGTTCTGTTCTTTTGTTCTC |
|  | Reverse | CTACGCTGACATTTTTTGAGGTTC |
| TFRC-M1A | Forward | CTCAGGAAGTGACGCACAGC |
|  | Reverse | TTCTAGAAGCCCGCACTCAC |
| GAPDH | Forward | TTCACCACCATGGAGAAGGC |
|  | Reverse | CTCGTGGTTCACACCCATCA |
